# Crystal structure and luminescence properties of 2-[(2′,6′-dimeth­oxy-2,3′-bipyridin-6-yl)­oxy]-9-(pyridin-2-yl)-9*H*-carbazole

**DOI:** 10.1107/S2056989019013549

**Published:** 2019-10-22

**Authors:** Suk-Hee Moon, Ki-Min Park, Jinho Kim, Youngjin Kang

**Affiliations:** a Department of Food and Nutrition, Kyungnam College of Information and Technology, Busan 47011, Republic of Korea; b Research Institute of Natural Science, Gyeongsang National University, Jinju 52828, Republic of Korea; c Division of Science Education & Department of Chemistry, Kangwon National University, Chuncheon 24341, Republic of Korea

**Keywords:** crystal structure, carbazole derivative, hydrogen bonds, π–π stacking inter­actions, luminescence

## Abstract

In the title com­pound, C_29_H_22_N_4_O_3_, the carbazole system forms a dihedral angle of 68.45 (3)° with the mean plane of the bi­pyridine ring system. The bi­pyridine ring system, with two meth­oxy substituents, is approximately planar (r.m.s. deviation = 0.0670 Å), with a dihedral angle of 7.91 (13)° between the planes of the two pyridine rings. Intra­molecular C—H⋯O/N hydrogen bonds may promote the planarity of the bipyridyl ring system. In the pyridyl-substituted carbazole fragment, the pyridine ring is tilted by 56.65 (4)° with respect to the mean plane of the carbazole system (r.m.s. deviation = 0.0191 Å). In the crystal, adjacent mol­ecules are connected *via* C—H⋯O/N hydrogen bonds and C—H⋯π inter­actions, resulting in the formation of a three-dimensional (3D) supra­molecular network. In addition, the 3D structure contains inter­molecular π–π stacking inter­actions, with centroid–centroid distances of 3.5634 (12) Å between pyridine rings. The title com­pound exhibits a high energy gap (3.48 eV) and triplet energy (2.64 eV), indicating that it could be a suitable host material in organic light-emitting diode (OLED) applications.

## Abstract 2

In the title com­pound, the dihedral angle between the planes of the bi­pyridine and carbazole moieties connected by an O atom is 68.45 (3)°. The bi­pyridine ring system, with two meth­oxy substituents, is approximately planar. The pyridine ring in the pyridyl-substituted carbazole fragment is tilted by 56.65 (4)° with respect to the mean plane of the carbazole moiety. The title com­pound exhibits a high energy gap and triplet energy.

## Chemical context   

Carbazole-based organic small mol­ecules have recently attracted much inter­est as organic light-emitting diodes (OLEDs) because of their high stability to the redox process, as well as their high triplet energy (*E*_T_ ≃ 3.0 eV) (Krucaite & Grigalevicius, 2019 ▸). In particular, organic com­pounds bearing a carbazole group have been widely used as host materials for phospho­rescent organic light-emitting diodes (PhOLEDs) due to their high thermal stability and excellent hole-transporting properties (Yang *et al.*, 2018 ▸). Moreover, a number of carbazole-based com­pounds have been developed as ligands to coordinate with heavy transition-metal ions, such as Pd^II^ and Pt^II^ (Fleetham *et al.*, 2017 ▸). Although there are a number of carbazole-based organic com­pounds, examples linking a bi­pyridine functional group to a carbazole unit are still rare. Based on previous reports, bi­pyridine also possesses a high triplet energy and a stable chelated coordination mode with respect to transition-metal ions, which makes it a suitable ligand for developing blue phospho­rescent metal com­plexes (Zaen *et al.*, 2019 ▸). Despite this advantage, reports of crystal structures of carbazole derivatives are still scarce. This prompted us to investigate the crystal structure of carbazole derivatives bearing the bi­pyridine group. Herein, we describe the mol­ecular and crystal structures of 2-[(2′,6′-dimeth­oxy-2,3′-bipyridin-6-yl)­oxy]-9-(pyridin-2-yl)-9*H*-carbazole, which can act as a potential tetra­dentate ligand for various transition-metal ions. In addition, the luminescence properties of the title com­pound were examined *via* photophysical analysis.
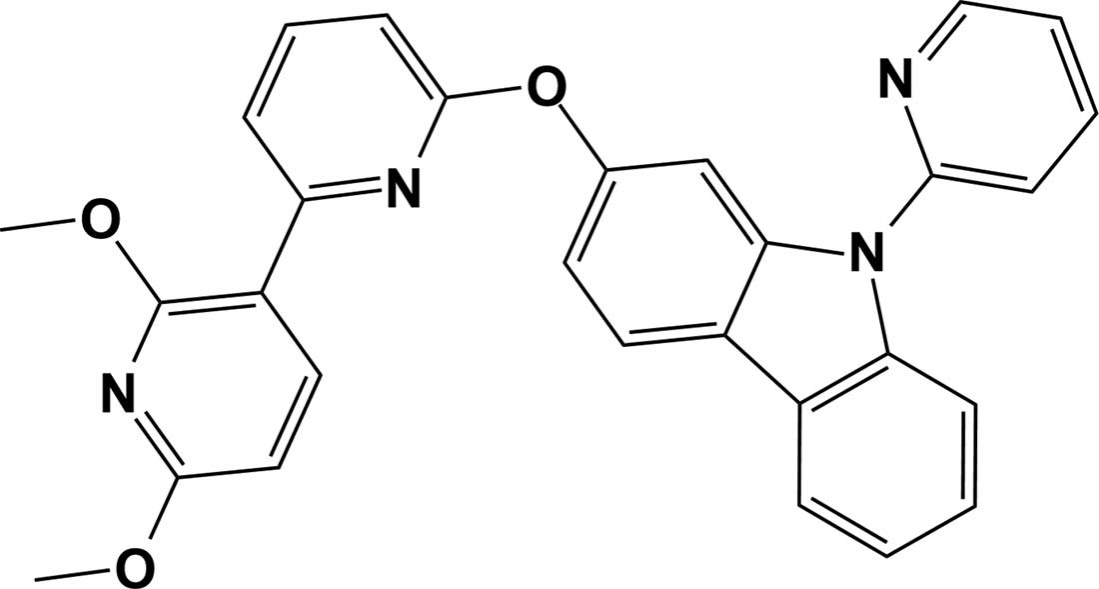


## Structural commentary   

**Figure 1:**
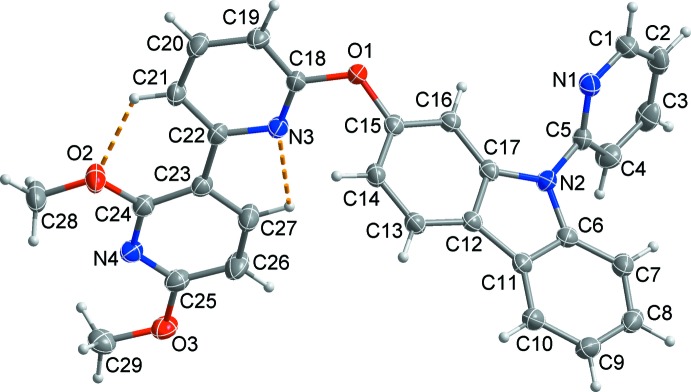
A view of the mol­ecular structure of the title com­pound, showing the atom-numbering scheme. Displacement ellipsoids are drawn at the 50% probability level. H atoms are presented as small spheres of arbitrary radius and yellow dashed lines represent intra­molecular C—H⋯O contacts.

Fig. 1 ▸ illustrates the mol­ecular structure of the title com­pound, in which the dihedral angle between the planes of the bi­pyridine (N3/C18–C27/N4) and carbazole (N2/C6–C17) moieties connected by atom O1 is 68.45 (3)°. In the pyridyl-substituted carbazole unit, the pyridine ring (N1/C1–C5) forms a dihedral angle of 56.65 (4)° with the carbazole ring system. The two pyridine rings in the bi­pyridine ring system, with two meth­oxy substituents, are approximately coplanar, making a dihedral angle of 7.91 (13)°. Short intra­molecular C—H⋯O and C—H⋯N contacts (Table 1 ▸), forming *S*(6) and *S*(5) rings, respectively, may contribute to the planarity of the bipyridyl ring system (r.m.s. deviation = 0.0670 Å).

## Supra­molecular features   

**Figure 2:**
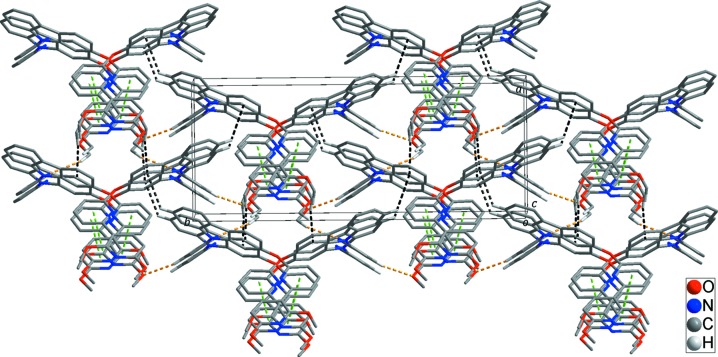
The 3D supra­molecular network formed through inter­molecular C—H⋯O/N hydrogen bonds (yellow dashed lines), C—H⋯π inter­actions (black dashed lines) and π–π stacking inter­actions (green dashed lines). H atoms not involved in the inter­molecular inter­actions have been omitted for clarity.

In the crystal, adjacent mol­ecules are connected by weak C—H⋯O/N hydrogen bonds and C—H⋯π inter­actions (Table 1 ▸ and yellow and black dashed lines in Fig. 2 ▸), forming a three-dimensional (3D) supra­molecular network. In addition, the 3D structure is stabilized by π–π stacking inter­actions (green dashed lines in Fig. 2 ▸), with a centroid–centroid distance of 3.5634 (12) Å for *Cg*3⋯*Cg*4(*x*, −*y* + 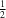, *z* − 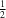), where *Cg*3 and *Cg*4 are the centroids of the N3- and N4-containing pyridine rings, respectively.

## Luminescence properties   

**Figure 3:**
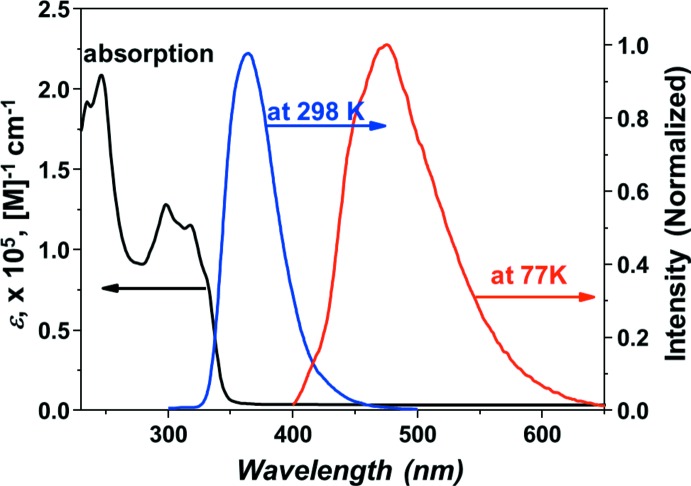
UV–Vis absorption and photoluminescence spectra of the title com­pound in CH_2_Cl_2_ solution.

The photophysical properties of the title com­pound were analyzed using UV–Vis and photoluminescence (PL) measurements. Fig. 3 ▸ shows the absorption, solution PL and low-temperature (77 K) PL spectra of the title com­pound. The com­pound showed a strong absorption of the carbazole unit above 300 nm and of the bi­pyridine unit connected to carbazole below 300 nm (Belletête *et al.*, 2004 ▸). The emission spectra were obtained under excitation at 280 nm. The title com­pound displays a narrow emission band, with λ_max_ = 364 nm, at ambient temperature. However, a broad emission, with λ_max_ = 470 nm, was observed at 77 K. The energy difference between the vibrationally relaxed ground and excited states, *E*_0–0_, which is defined as the crossing point of the appropriate absorption and emission spectra, is approximately 3.68 eV. The absorption edge of the UV–Vis spectrum was 356 nm, which corresponded to an energy gap at 3.48 eV. The triplet energy of the title com­pound was 2.64 eV, which could be calculated from the phospho­rescent emission maximum (470 nm) of the PL spectrum at 77 K. This value was high enough to suggest the use of the host material as a green phospho­rescent dopant. The triplet energy of the tris­(2-phenyl­pyridinato-κ^2^ *C* ^2^,*N*)iridium(III), or Ir(ppy)_3_, dopant is 2.40 eV and effective energy transfer from the title com­pound to the Ir(ppy)_3_ dopant is expected. Consequently, strong absorption and a high energy gap and triplet energy make the title com­pound a suitable host material in organic light-emitting diode (OLED) applications.

## Synthesis and crystallization   

**Figure 4:**
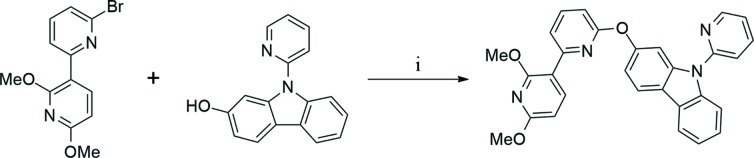
Synthetic route and reagents to obtain the title com­pound: (i) CuI (0.1 equiv.), picolinic acid (0.2 equiv.), K_3_PO_4_ (2 equiv.) and DMSO; 373 K and 72 h.

All experiments were performed under a dry N_2_ atmosphere using standard Schlenk techniques. All solvents used in this study were freshly distilled over appropriate drying reagents prior to use. All starting materials were commercially purchased and used without further purification. The ^1^H NMR spectrum was recorded on a JEOL 400 MHz spectrometer. The two starting materials, *i.e.* 6-bromo-2′,6′-dimeth­oxy-2,3′-bi­pyridine and 9-(pyridin-2-yl)-9*H*-carbazol-2-ol, were syn­the­sized according to a slight modification of a previous synthetic methodology reported by our group (Park *et al.*, 2018 ▸; Fleetham *et al.*, 2016 ▸). Details of the synthetic procedures and reagents are presented in Fig. 4 ▸.

To a 100 ml Schlenk flask were added 9-(pyridin-2-yl)-9*H*-carbazol-2-ol (1.0 g, 3.84 mmol), 6-bromo-2′,6′-dimeth­oxy-2,3′-bi­pyridine (1.36 g, 4.61 mmol), CuI (0.073 mg, 0.384 mmol), 2-picolinic acid (0.094 g, 0.758 mmol) and K_3_PO_4_ (1.63 g, 7.68 mmol). The flask was evacuated and backfilled with nitro­gen and then dimethyl sulfoxide (DMSO; 15 ml) was then added under an N_2_ atmosphere. The reaction mixture was stirred at 368–378 K still under nitro­gen for 3 d. After cooling to room temperature, the mixture was poured into water (100 ml) and extracted with ethyl acetate (50 ml × 3). The combined organic layer was dried with anhydrous Na_2_SO_4_ and concentrated under reduced pressure. Purification by column chromatography (di­chloro­methane–hexane 1:10 and then 1:3 *v*/*v*) afforded the desired product as a white solid (yield 1.3 g, 72%). Colourless crystals of X-ray quality were obtained by slow evaporation of a di­chloro­methane–hexane solution (1:1 *v*/*v*) of the title com­pound. ^1^H NMR (400 MHz, CDCl_3_): δ 8.66 (*ddd*, *J* = 5.6, 2.0, 0.8 Hz, 1H), 8.21 (*d*, *J* = 8.0 Hz, 1H), 8.08 (*s*, 1H), 8.01 (*s*, 1H), 7.86 (*td*, *J* = 7.6, 1.6 Hz, 1H), 7.82 (*d*, *J* = 8.4 Hz, 1H), 7.79 (*d*, *J* = 8.0 Hz, 1H), 7.72 (*d*, *J* = 2.0 Hz, 1H), 7.67 (*t*, *J* = 8.0 Hz, 1H), 7.62 (*dd*, *J* = 8.0, 0.8 Hz, 1H), 7.42 (*td*, *J* = 7.6, 1.2 Hz, 1H), 7.32 (*td*, *J* = 7.6, 0.8 Hz, 1H), 7.27 (*td*, *J* = 5.0, 1.2 Hz, 1H), 7.15 (*dd*, *J* = 7.6, 1.6 Hz, 1H), 6.71 (*d*, *J* = 8.4 Hz, 1H), 6.30 (*d*, *J* = 8.8 Hz, 1H), 4.02 (*s*, 3H), 3.93 (*s*, 3H); ^13^C NMR (100 MHz, CDCl_3_): δ 163.4, 163.0, 160.1, 153.2, 152.2, 151.7, 149.7, 142.4, 140.4, 140.0, 139.8, 138.7, 125.9, 124.2, 121.5, 121.3, 121.2, 120.9, 120.0, 119.1, 118.3, 115.1, 113.4, 111.2, 108.3, 104.4, 101.8, 53.8, 53.6; HRMS (EI): found *m*/*z* 474.

## Refinement   

Crystal data, data collection and structure refinement details are summarized in Table 2 ▸. All H atoms were positioned geometrically and refined using a riding model, with C—H = 0.95 Å and *U*_iso_(H) = 1.2*U*_eq_(C) for C*sp* ^2^ H atoms, and with C—H = 0.98 Å and *U*_iso_(H) = 1.5*U*_eq_(C) for methyl H atoms.

## Supplementary Material

Crystal structure: contains datablock(s) I, New_Global_Publ_Block. DOI: 10.1107/S2056989019013549/fy2142sup1.cif

Structure factors: contains datablock(s) I. DOI: 10.1107/S2056989019013549/fy2142Isup2.hkl

CCDC references: 1957451, 1957451

Additional supporting information:  crystallographic information; 3D view; checkCIF report

## Figures and Tables

**Table 1:** Hydrogen-bond geometry (Å, °) *Cg*1 is the centroid of the C12–C17 ring.

*D*—H⋯*A*	*D*—H	H⋯*A*	*D*⋯*A*	*D*—H⋯*A*
C3—H3⋯O3^i^	0.95	2.50	3.429 (3)	165
C21—H21⋯O2	0.95	2.20	2.839 (3)	123
C27—H27⋯N3	0.95	2.35	2.720 (3)	102
C29—H29*A*⋯N1^ii^	0.98	2.54	3.442 (3)	154
C8—H8⋯*Cg*1^iii^	0.95	2.76	3.426 (2)	128
C28—H28*C*⋯*Cg*1^iv^	0.98	2.89	3.485 (3)	120

Symmetry codes: (i) 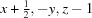; (ii) 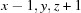; (iii) 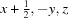; (iv) 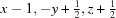.

**Table 2:** Experimental details

Crystal data	
Chemical formula	C_29_H_22_N_4_O_3_
*M*_r_	474.50
Crystal system, space group	Monoclinic, *I* *a*
Temperature (K)	173
*a*, *b*, *c* (Å)	9.6979 (1), 23.6702 (3), 9.9229 (2)
β (°)	92.9125 (5)
*V* (Å^3^)	2274.87 (6)
*Z*	4
Radiation type	Mo *K*α
μ (mm^−1^)	0.09
Crystal size (mm)	0.53 × 0.46 × 0.12

Data collection	
Diffractometer	Bruker APEXII CCD
Absorption correction	Multi-scan (*SADABS*; Bruker, 2014 ▸)
*T*_min_, *T*_max_	0.710, 0.746
No. of measured, independent and observed [*I* > 2σ(*I*)] reflections	10918, 5179, 4943
*R*_int_	0.020
(sin θ/λ)_max_ (Å^−1^)	0.669

Refinement	
*R*[*F* ^2^ > 2σ(*F* ^2^)], *wR*(*F* ^2^), *S*	0.034, 0.084, 1.06
No. of reflections	5179
No. of parameters	328
No. of restraints	2
H-atom treatment	H-atom parameters constrained
Δρ_max_, Δρ_min_ (e Å^−3^)	0.21, −0.19

Computer programs: *APEX2* (Bruker, 2014 ▸), *SAINT* (Bruker, 2014 ▸), *SHELXS97* (Sheldrick, 2008 ▸), *SHELXL2014* (Sheldrick, 2015 ▸), *DIAMOND* (Brandenburg, 2010 ▸), *SHELXTL* (Sheldrick, 2008 ▸) and *publCIF* (Westrip, 2010 ▸).

## supplementary crystallographic information

### Crystal data

**Table:**

C_29_H_22_N_4_O_3_	*F*(000) = 992
*M**_r_* = 474.50	*D*_x_ = 1.385 Mg m^−^^3^
Monoclinic, *I**a*	Mo *K*α radiation, λ = 0.71073 Å
*a* = 9.6979 (1) Å	Cell parameters from 6053 reflections
*b* = 23.6702 (3) Å	θ = 2.2–28.3°
*c* = 9.9229 (2) Å	µ = 0.09 mm^−^^1^
β = 92.9125 (5)°	*T* = 173 K
*V* = 2274.87 (6) Å^3^	Block, colourless
*Z* = 4	0.53 × 0.46 × 0.12 mm

### Data collection

**Table:**

Bruker APEXII CCD diffractometer	4943 reflections with *I* > 2σ(*I*)
φ and ω scans	*R*_int_ = 0.020
Absorption correction: multi-scan (SADABS; Bruker, 2014)	θ_max_ = 28.4°, θ_min_ = 1.7°
*T*_min_ = 0.710, *T*_max_ = 0.746	*h* = −12→12
10918 measured reflections	*k* = −31→31
5179 independent reflections	*l* = −13→12

### Refinement

**Table:**

Refinement on *F*^2^	Secondary atom site location: difference Fourier map
Least-squares matrix: full	Hydrogen site location: inferred from neighbouring sites
*R*[*F*^2^ > 2σ(*F*^2^)] = 0.034	H-atom parameters constrained
*wR*(*F*^2^) = 0.084	*w* = 1/[σ^2^(*F*_o_^2^) + (0.0404*P*)^2^ + 0.7038*P*] where *P* = (*F*_o_^2^ + 2*F*_c_^2^)/3
*S* = 1.06	(Δ/σ)_max_ < 0.001
5179 reflections	Δρ_max_ = 0.21 e Å^−^^3^
328 parameters	Δρ_min_ = −0.19 e Å^−^^3^
2 restraints	Extinction correction: SHELXL2014 (Sheldrick, 2015), Fc^*^=kFc[1+0.001xFc^2^λ^3^/sin(2θ)]^-1/4^
Primary atom site location: structure-invariant direct methods	Extinction coefficient: 0.0062 (6)

### Special details

**Table:**

Geometry. All esds (except the esd in the dihedral angle between two l.s. planes) are estimated using the full covariance matrix. The cell esds are taken into account individually in the estimation of esds in distances, angles and torsion angles; correlations between esds in cell parameters are only used when they are defined by crystal symmetry. An approximate (isotropic) treatment of cell esds is used for estimating esds involving l.s. planes.

### Fractional atomic coordinates and isotropic or equivalent isotropic displacement parameters (Å^2^)

**Table:**

	*x*	*y*	*z*	*U*_iso_*/*U*_eq_	
O1	0.16676 (16)	0.24337 (6)	0.28067 (15)	0.0285 (3)	
O2	−0.27313 (17)	0.33138 (6)	0.70330 (18)	0.0382 (4)	
O3	−0.43670 (17)	0.15756 (6)	0.82051 (17)	0.0358 (4)	
N1	0.3339 (2)	0.06572 (7)	0.02064 (19)	0.0287 (4)	
N2	0.30895 (18)	0.04679 (7)	0.24867 (18)	0.0270 (4)	
N3	−0.00057 (17)	0.25161 (7)	0.43700 (18)	0.0244 (3)	
N4	−0.35398 (18)	0.24417 (7)	0.75714 (19)	0.0273 (4)	
C1	0.3152 (3)	0.05001 (10)	−0.1083 (2)	0.0344 (5)	
H1	0.3516	0.0737	−0.1752	0.041*	
C2	0.2465 (3)	0.00167 (10)	−0.1504 (2)	0.0360 (5)	
H2	0.2399	−0.0086	−0.2431	0.043*	
C3	0.1874 (3)	−0.03137 (10)	−0.0545 (3)	0.0379 (5)	
H3	0.1378	−0.0646	−0.0801	0.045*	
C4	0.2016 (3)	−0.01536 (9)	0.0794 (2)	0.0353 (5)	
H4	0.1594	−0.0365	0.1475	0.042*	
C5	0.2793 (2)	0.03247 (8)	0.1115 (2)	0.0246 (4)	
C6	0.3774 (2)	0.01129 (8)	0.3428 (2)	0.0261 (4)	
C7	0.4216 (2)	−0.04424 (9)	0.3285 (2)	0.0305 (4)	
H7	0.4050	−0.0642	0.2462	0.037*	
C8	0.4904 (2)	−0.06934 (9)	0.4381 (2)	0.0331 (5)	
H8	0.5208	−0.1073	0.4311	0.040*	
C9	0.5164 (3)	−0.04006 (10)	0.5592 (2)	0.0356 (5)	
H9	0.5633	−0.0584	0.6333	0.043*	
C10	0.4742 (2)	0.01540 (9)	0.5717 (2)	0.0319 (5)	
H10	0.4937	0.0355	0.6534	0.038*	
C11	0.4030 (2)	0.04161 (8)	0.4635 (2)	0.0253 (4)	
C12	0.3469 (2)	0.09767 (8)	0.44159 (19)	0.0231 (4)	
C13	0.3371 (2)	0.14504 (8)	0.5239 (2)	0.0259 (4)	
H13	0.3741	0.1443	0.6144	0.031*	
C14	0.2730 (2)	0.19313 (8)	0.4724 (2)	0.0263 (4)	
H14	0.2648	0.2256	0.5277	0.032*	
C15	0.2203 (2)	0.19380 (8)	0.3386 (2)	0.0243 (4)	
C16	0.2283 (2)	0.14802 (8)	0.2536 (2)	0.0240 (4)	
H16	0.1920	0.1493	0.1629	0.029*	
C17	0.2923 (2)	0.09974 (8)	0.3073 (2)	0.0232 (4)	
C18	0.0820 (2)	0.27662 (8)	0.3548 (2)	0.0239 (4)	
C19	0.0883 (2)	0.33456 (9)	0.3325 (2)	0.0309 (5)	
H19	0.1491	0.3506	0.2708	0.037*	
C20	0.0015 (2)	0.36732 (9)	0.4049 (2)	0.0347 (5)	
H20	0.0019	0.4072	0.3941	0.042*	
C21	−0.0865 (2)	0.34265 (9)	0.4935 (2)	0.0299 (5)	
H21	−0.1461	0.3653	0.5440	0.036*	
C22	−0.0865 (2)	0.28402 (8)	0.5075 (2)	0.0238 (4)	
C23	−0.1789 (2)	0.25192 (8)	0.5941 (2)	0.0251 (4)	
C24	−0.2697 (2)	0.27479 (8)	0.6851 (2)	0.0261 (4)	
C25	−0.3517 (2)	0.18857 (9)	0.7446 (2)	0.0301 (4)	
C26	−0.2677 (3)	0.16054 (10)	0.6591 (3)	0.0431 (6)	
H26	−0.2689	0.1205	0.6519	0.052*	
C27	−0.1815 (3)	0.19309 (9)	0.5844 (3)	0.0374 (5)	
H27	−0.1222	0.1749	0.5245	0.045*	
C28	−0.3786 (3)	0.35291 (10)	0.7870 (3)	0.0458 (6)	
H28A	−0.3613	0.3930	0.8061	0.069*	
H28B	−0.3770	0.3317	0.8719	0.069*	
H28C	−0.4693	0.3486	0.7398	0.069*	
C29	−0.5206 (3)	0.18868 (10)	0.9090 (3)	0.0366 (5)	
H29A	−0.5724	0.1623	0.9634	0.055*	
H29B	−0.5854	0.2125	0.8555	0.055*	
H29C	−0.4616	0.2125	0.9686	0.055*	

### Atomic displacement parameters (Å^2^)

**Table:**

	*U*^11^	*U*^22^	*U*^33^	*U*^12^	*U*^13^	*U*^23^
O1	0.0366 (8)	0.0230 (7)	0.0266 (7)	0.0077 (6)	0.0087 (6)	0.0039 (6)
O2	0.0429 (9)	0.0230 (7)	0.0510 (10)	−0.0008 (6)	0.0243 (8)	−0.0039 (7)
O3	0.0361 (8)	0.0275 (8)	0.0446 (10)	−0.0009 (6)	0.0100 (8)	0.0102 (7)
N1	0.0347 (9)	0.0247 (8)	0.0272 (9)	−0.0012 (7)	0.0062 (7)	−0.0023 (7)
N2	0.0367 (10)	0.0195 (8)	0.0249 (9)	0.0020 (7)	0.0032 (7)	−0.0003 (6)
N3	0.0248 (8)	0.0212 (8)	0.0275 (8)	0.0015 (6)	0.0032 (7)	0.0005 (6)
N4	0.0247 (9)	0.0268 (8)	0.0306 (9)	0.0009 (7)	0.0032 (7)	0.0034 (7)
C1	0.0435 (13)	0.0330 (11)	0.0272 (11)	0.0016 (10)	0.0085 (9)	−0.0003 (9)
C2	0.0410 (13)	0.0362 (12)	0.0303 (11)	0.0063 (10)	−0.0024 (10)	−0.0106 (9)
C3	0.0409 (13)	0.0272 (11)	0.0448 (14)	−0.0027 (9)	−0.0062 (11)	−0.0101 (9)
C4	0.0420 (13)	0.0277 (11)	0.0364 (12)	−0.0074 (9)	0.0034 (10)	−0.0005 (9)
C5	0.0278 (10)	0.0206 (9)	0.0254 (10)	0.0023 (7)	0.0025 (8)	−0.0029 (7)
C6	0.0282 (10)	0.0245 (9)	0.0262 (10)	0.0008 (8)	0.0072 (8)	0.0032 (7)
C7	0.0367 (11)	0.0240 (9)	0.0314 (11)	0.0010 (8)	0.0094 (9)	−0.0002 (8)
C8	0.0342 (11)	0.0248 (10)	0.0413 (12)	0.0050 (8)	0.0116 (9)	0.0054 (9)
C9	0.0386 (12)	0.0349 (11)	0.0337 (12)	0.0095 (9)	0.0047 (10)	0.0104 (9)
C10	0.0346 (11)	0.0351 (11)	0.0265 (10)	0.0070 (9)	0.0055 (9)	0.0045 (8)
C11	0.0247 (10)	0.0254 (9)	0.0264 (10)	0.0022 (7)	0.0078 (8)	0.0023 (8)
C12	0.0227 (9)	0.0248 (9)	0.0222 (9)	0.0009 (7)	0.0048 (7)	0.0019 (7)
C13	0.0276 (9)	0.0288 (10)	0.0215 (9)	0.0012 (8)	0.0034 (7)	−0.0012 (8)
C14	0.0291 (10)	0.0236 (9)	0.0268 (10)	0.0007 (8)	0.0064 (8)	−0.0039 (8)
C15	0.0238 (9)	0.0217 (9)	0.0283 (10)	0.0009 (7)	0.0088 (8)	0.0019 (7)
C16	0.0249 (9)	0.0243 (9)	0.0231 (9)	−0.0010 (7)	0.0043 (7)	0.0006 (7)
C17	0.0248 (9)	0.0220 (9)	0.0233 (9)	−0.0024 (7)	0.0058 (8)	−0.0015 (7)
C18	0.0256 (9)	0.0227 (9)	0.0235 (9)	0.0039 (8)	0.0016 (8)	−0.0004 (7)
C19	0.0330 (11)	0.0242 (10)	0.0364 (12)	0.0018 (8)	0.0099 (9)	0.0067 (8)
C20	0.0398 (12)	0.0203 (9)	0.0448 (13)	0.0048 (9)	0.0109 (10)	0.0036 (9)
C21	0.0297 (10)	0.0243 (10)	0.0364 (12)	0.0059 (8)	0.0096 (9)	−0.0003 (8)
C22	0.0223 (9)	0.0221 (9)	0.0269 (10)	0.0016 (7)	−0.0002 (8)	0.0006 (7)
C23	0.0226 (9)	0.0228 (9)	0.0300 (10)	0.0025 (7)	0.0018 (8)	0.0030 (8)
C24	0.0246 (9)	0.0242 (9)	0.0294 (10)	0.0011 (7)	0.0014 (8)	0.0016 (8)
C25	0.0270 (10)	0.0275 (10)	0.0359 (11)	−0.0003 (8)	0.0025 (9)	0.0070 (9)
C26	0.0481 (15)	0.0218 (10)	0.0613 (17)	0.0015 (10)	0.0208 (13)	0.0050 (10)
C27	0.0407 (12)	0.0241 (10)	0.0489 (14)	0.0037 (9)	0.0182 (11)	0.0004 (9)
C28	0.0540 (15)	0.0298 (11)	0.0565 (16)	0.0028 (11)	0.0298 (13)	−0.0055 (11)
C29	0.0359 (12)	0.0377 (12)	0.0370 (12)	−0.0085 (10)	0.0090 (10)	0.0034 (10)

### Geometric parameters (Å, º)

**Table:**

O1—C18	1.378 (2)	C10—H10	0.9500
O1—C15	1.395 (2)	C11—C12	1.446 (3)
O2—C24	1.352 (2)	C12—C13	1.393 (3)
O2—C28	1.443 (3)	C12—C17	1.409 (3)
O3—C25	1.360 (3)	C13—C14	1.383 (3)
O3—C29	1.431 (3)	C13—H13	0.9500
N1—C5	1.327 (3)	C14—C15	1.398 (3)
N1—C1	1.335 (3)	C14—H14	0.9500
N2—C17	1.395 (2)	C15—C16	1.378 (3)
N2—C6	1.399 (3)	C16—C17	1.393 (3)
N2—C5	1.417 (3)	C16—H16	0.9500
N3—C18	1.312 (3)	C18—C19	1.391 (3)
N3—C22	1.353 (3)	C19—C20	1.373 (3)
N4—C25	1.322 (3)	C19—H19	0.9500
N4—C24	1.328 (3)	C20—C21	1.386 (3)
C1—C2	1.379 (3)	C20—H20	0.9500
C1—H1	0.9500	C21—C22	1.395 (3)
C2—C3	1.379 (4)	C21—H21	0.9500
C2—H2	0.9500	C22—C23	1.482 (3)
C3—C4	1.382 (3)	C23—C27	1.396 (3)
C3—H3	0.9500	C23—C24	1.402 (3)
C4—C5	1.388 (3)	C25—C26	1.377 (3)
C4—H4	0.9500	C26—C27	1.380 (3)
C6—C7	1.392 (3)	C26—H26	0.9500
C6—C11	1.407 (3)	C27—H27	0.9500
C7—C8	1.380 (3)	C28—H28A	0.9800
C7—H7	0.9500	C28—H28B	0.9800
C8—C9	1.399 (3)	C28—H28C	0.9800
C8—H8	0.9500	C29—H29A	0.9800
C9—C10	1.382 (3)	C29—H29B	0.9800
C9—H9	0.9500	C29—H29C	0.9800
C10—C11	1.393 (3)		
			
C18—O1—C15	118.70 (15)	C16—C15—O1	116.16 (18)
C24—O2—C28	116.72 (17)	C16—C15—C14	122.88 (18)
C25—O3—C29	116.20 (17)	O1—C15—C14	120.73 (17)
C5—N1—C1	116.50 (19)	C15—C16—C17	116.67 (18)
C17—N2—C6	108.79 (16)	C15—C16—H16	121.7
C17—N2—C5	126.41 (16)	C17—C16—H16	121.7
C6—N2—C5	124.30 (16)	C16—C17—N2	129.49 (18)
C18—N3—C22	118.47 (17)	C16—C17—C12	121.93 (18)
C25—N4—C24	118.63 (19)	N2—C17—C12	108.51 (16)
N1—C1—C2	124.2 (2)	N3—C18—O1	118.27 (17)
N1—C1—H1	117.9	N3—C18—C19	125.21 (19)
C2—C1—H1	117.9	O1—C18—C19	116.49 (19)
C3—C2—C1	118.2 (2)	C20—C19—C18	116.1 (2)
C3—C2—H2	120.9	C20—C19—H19	121.9
C1—C2—H2	120.9	C18—C19—H19	121.9
C2—C3—C4	118.9 (2)	C19—C20—C21	120.5 (2)
C2—C3—H3	120.5	C19—C20—H20	119.7
C4—C3—H3	120.5	C21—C20—H20	119.7
C3—C4—C5	118.1 (2)	C20—C21—C22	119.04 (19)
C3—C4—H4	120.9	C20—C21—H21	120.5
C5—C4—H4	120.9	C22—C21—H21	120.5
N1—C5—C4	123.9 (2)	N3—C22—C21	120.63 (19)
N1—C5—N2	116.24 (18)	N3—C22—C23	114.54 (16)
C4—C5—N2	119.80 (19)	C21—C22—C23	124.80 (18)
C7—C6—N2	129.6 (2)	C27—C23—C24	114.82 (19)
C7—C6—C11	121.8 (2)	C27—C23—C22	118.74 (19)
N2—C6—C11	108.61 (17)	C24—C23—C22	126.41 (18)
C8—C7—C6	117.7 (2)	N4—C24—O2	116.67 (18)
C8—C7—H7	121.1	N4—C24—C23	124.05 (18)
C6—C7—H7	121.1	O2—C24—C23	119.27 (18)
C7—C8—C9	121.5 (2)	N4—C25—O3	118.2 (2)
C7—C8—H8	119.3	N4—C25—C26	123.4 (2)
C9—C8—H8	119.3	O3—C25—C26	118.41 (19)
C10—C9—C8	120.4 (2)	C25—C26—C27	117.1 (2)
C10—C9—H9	119.8	C25—C26—H26	121.4
C8—C9—H9	119.8	C27—C26—H26	121.4
C9—C10—C11	119.4 (2)	C26—C27—C23	121.9 (2)
C9—C10—H10	120.3	C26—C27—H27	119.0
C11—C10—H10	120.3	C23—C27—H27	119.0
C10—C11—C6	119.22 (19)	O2—C28—H28A	109.5
C10—C11—C12	133.8 (2)	O2—C28—H28B	109.5
C6—C11—C12	106.95 (17)	H28A—C28—H28B	109.5
C13—C12—C17	119.52 (18)	O2—C28—H28C	109.5
C13—C12—C11	133.37 (18)	H28A—C28—H28C	109.5
C17—C12—C11	107.10 (17)	H28B—C28—H28C	109.5
C14—C13—C12	119.24 (19)	O3—C29—H29A	109.5
C14—C13—H13	120.4	O3—C29—H29B	109.5
C12—C13—H13	120.4	H29A—C29—H29B	109.5
C13—C14—C15	119.76 (18)	O3—C29—H29C	109.5
C13—C14—H14	120.1	H29A—C29—H29C	109.5
C15—C14—H14	120.1	H29B—C29—H29C	109.5
			
C5—N1—C1—C2	−1.4 (3)	C6—N2—C17—C16	−178.7 (2)
N1—C1—C2—C3	3.2 (4)	C5—N2—C17—C16	9.2 (3)
C1—C2—C3—C4	−1.1 (4)	C6—N2—C17—C12	−1.7 (2)
C2—C3—C4—C5	−2.3 (4)	C5—N2—C17—C12	−173.88 (18)
C1—N1—C5—C4	−2.4 (3)	C13—C12—C17—C16	0.0 (3)
C1—N1—C5—N2	175.06 (19)	C11—C12—C17—C16	179.17 (18)
C3—C4—C5—N1	4.3 (3)	C13—C12—C17—N2	−177.21 (18)
C3—C4—C5—N2	−173.1 (2)	C11—C12—C17—N2	2.0 (2)
C17—N2—C5—N1	51.1 (3)	C22—N3—C18—O1	−178.41 (17)
C6—N2—C5—N1	−119.9 (2)	C22—N3—C18—C19	−0.6 (3)
C17—N2—C5—C4	−131.3 (2)	C15—O1—C18—N3	−34.5 (3)
C6—N2—C5—C4	57.7 (3)	C15—O1—C18—C19	147.49 (19)
C17—N2—C6—C7	−177.0 (2)	N3—C18—C19—C20	0.9 (3)
C5—N2—C6—C7	−4.6 (3)	O1—C18—C19—C20	178.79 (19)
C17—N2—C6—C11	0.8 (2)	C18—C19—C20—C21	−0.4 (4)
C5—N2—C6—C11	173.16 (18)	C19—C20—C21—C22	−0.4 (4)
N2—C6—C7—C8	178.5 (2)	C18—N3—C22—C21	−0.3 (3)
C11—C6—C7—C8	1.0 (3)	C18—N3—C22—C23	178.03 (17)
C6—C7—C8—C9	−0.6 (3)	C20—C21—C22—N3	0.8 (3)
C7—C8—C9—C10	−0.5 (4)	C20—C21—C22—C23	−177.4 (2)
C8—C9—C10—C11	1.3 (4)	N3—C22—C23—C27	−7.2 (3)
C9—C10—C11—C6	−1.0 (3)	C21—C22—C23—C27	171.1 (2)
C9—C10—C11—C12	−179.1 (2)	N3—C22—C23—C24	174.66 (18)
C7—C6—C11—C10	−0.2 (3)	C21—C22—C23—C24	−7.0 (3)
N2—C6—C11—C10	−178.16 (18)	C25—N4—C24—O2	−178.53 (19)
C7—C6—C11—C12	178.39 (19)	C25—N4—C24—C23	1.0 (3)
N2—C6—C11—C12	0.4 (2)	C28—O2—C24—N4	−6.9 (3)
C10—C11—C12—C13	−4.2 (4)	C28—O2—C24—C23	173.6 (2)
C6—C11—C12—C13	177.6 (2)	C27—C23—C24—N4	−0.6 (3)
C10—C11—C12—C17	176.8 (2)	C22—C23—C24—N4	177.60 (19)
C6—C11—C12—C17	−1.4 (2)	C27—C23—C24—O2	178.9 (2)
C17—C12—C13—C14	0.4 (3)	C22—C23—C24—O2	−2.9 (3)
C11—C12—C13—C14	−178.5 (2)	C24—N4—C25—O3	178.91 (18)
C12—C13—C14—C15	−0.6 (3)	C24—N4—C25—C26	−1.0 (4)
C18—O1—C15—C16	141.73 (19)	C29—O3—C25—N4	−0.6 (3)
C18—O1—C15—C14	−43.6 (3)	C29—O3—C25—C26	179.2 (2)
C13—C14—C15—C16	0.3 (3)	N4—C25—C26—C27	0.5 (4)
C13—C14—C15—O1	−174.02 (18)	O3—C25—C26—C27	−179.4 (2)
O1—C15—C16—C17	174.71 (16)	C25—C26—C27—C23	−0.1 (4)
C14—C15—C16—C17	0.1 (3)	C24—C23—C27—C26	0.1 (4)
C15—C16—C17—N2	176.29 (19)	C22—C23—C27—C26	−178.2 (2)
C15—C16—C17—C12	−0.3 (3)		

### Hydrogen-bond geometry (Å, º)

Cg1 is the centroid of the C12–C17 ring.

**Table:**

*D*—H···*A*	*D*—H	H···*A*	*D*···*A*	*D*—H···*A*
C3—H3···O3^i^	0.95	2.50	3.429 (3)	165
C21—H21···O2	0.95	2.20	2.839 (3)	123
C27—H27···N3	0.95	2.35	2.720 (3)	102
C29—H29*A*···N1^ii^	0.98	2.54	3.442 (3)	154
C8—H8···*Cg*1^iii^	0.95	2.76	3.426 (2)	128
C28—H28*C*···*Cg*1^iv^	0.98	2.89	3.485 (3)	120

Symmetry codes: (i) *x*+1/2, −*y*, *z*−1; (ii) *x*−1, *y*, *z*+1; (iii) *x*+1/2, −*y*, *z*; (iv) *x*−1, −*y*+1/2, *z*+1/2.
